# Current State of *Ammophila arenaria* (Marram Grass) Distribution in the Eastern Cape, South Africa, and the Possible Effect of the Grass on the Dune System Dynamics

**DOI:** 10.3390/plants11172260

**Published:** 2022-08-30

**Authors:** Roy A. Lubke

**Affiliations:** Department of Botany, Rhodes University, Grahamstown 6140, South Africa; r.lubke@ru.ac.za

**Keywords:** coastal management, dune systems, invasive alien plants, marram grass

## Abstract

The principal aim of this paper is to show that marram grass is not an invasive alien in South Africa although it affects the dune dynamics as a useful pioneer species in the dune successional process. The historical perspective of marram introduction as a dune stabiliser and the studies and conclusions reached from our European Union funded project, *INVASS*, in the 1990s and early 2000s is presented. Although these studies showed that marram was non-invasive, this was not clearly carried through to the authorities, and the use of the grass as a dune stabiliser was limited without a special permit. This prompted a survey of the current situation of marram on dune sites in the Eastern Cape. Along with earlier (1980s) data on the dunes, 69 relevés with 66 species abundance from sites along the Eastern Cape shoreline were assembled. These data were analysed with Detrended Correspondence Analysis to show the relationships of the samples (relevés) and species in a 2-dimensional scatter diagram. The survey showed that there are four dune sites where marram grass is no longer present, due to either marram being out of its climatic range, erosion of sand under storm conditions which made the habitat unsuitable, or in one case where marram simply disappeared. Marram often remains in other sites where three to five dune pioneer species were recorded. On some dunes, although marram is the most abundant dune pioneer, it is never dominant in the dune environment but has a presence of as much as 75% at any site. The eight pioneer species are widely dispersed on the DCA scatter diagram, while the shrub species characterising the Coastal Scrub are tightly clustered, showing that all the pioneer dune communities behave similarly in the dune successional series. The conclusion from these studies is that marram grass does not always persist in the dune systems. If marram does persist, it does not compete and behaves identically to the indigenous species as a dune pioneer. These studies show that marram grass is a non-invasive species that can be successfully used in dune stabilisation on Cape dunes.

## 1. Introduction

In Europe *Ammophila arenaria* (L.) Link. (marram grass) is the primary dune stabiliser of mobile dunes and is used extensively in the Netherlands, for example, in establishing barriers to the North Sea in that low-lying country [1]. In the 1880s, with the Cape’s connection with European countries, marram was an obvious choice to use as a stabilisation of foredunes and drift sands on the Cape Flats [2]. The Department of Forestry, who had jurisdiction of the coastal zone until the 1990s, planted hundreds of hectares throughout the mobile dunes of the then Cape Province (Table 1 shows the planting for the Eastern Cape). Later the coastal state lands were handed over to South African National Parks (SANParks) and the provincial conservation bodies. Because of a lack of understanding of dune ecology which was little researched in South Africa until the 1970s and 1980s, the Department of Forestry had a policy of stabilisation of all mobile dune systems that they incorrectly believed to be unnatural, having resulted due to human disturbance of the natural vegetation. Many non-indigenous species, especially invasive Australian *Acacia* spp., were introduced for dune stabilisation [3,4].

Marram grass is aggressively invasive in some countries [1,5]. At the International Dunes Conference in Port Elizabeth in 1994, Wiedemann [6] pointed out the problems of marram grass invasion of the west coast of the USA [7]. 

Consequently, some 25 years ago, Lubke and Hertling initiated a research project on the grass and published a paper that questioned whether marram grass was a potential invader in the Cape as it is on the west Coast of North America [8]. The purpose of this paper is to state categorically that our assumption was wrong. We explain what led us to this conclusion and how we established that marram grass is not an invasive alien grass, and how it may affect the dune dynamics as a useful pioneer, similar to indigenous pioneers.

### 1.1. Investigations into the Potential of Marram Grass as an Invasive Species 

Because of the problems of the invasiveness of other dune stabilisation species such as *Acacia saligna* (Port Jackson wattle) and *Acacia cyclops* (rooikrans), marram grass was also included in this category especially as we had asked the question whether it was an invasive species [9]. This was a prelude to an extensive survey of the extent of its use and its potential to invade coastal sands in the Eastern and Western Cape as it does in other countries. Van der Putten [5] suggests that a combination of factors, which include the enemy release, biotic resistance, and accumulation of local pathogens hypothesis, are the reasons why marram grass is or can be invasive in its non-native range. Consequently, we initiated a large European Union-funded study at Rhodes University (*INVASS*) on potentially invasive grass species with colleagues in Botswana, UK, and the Netherlands (see list of references in Table 2 on some of these studies). In addition to the intensive studies of marram populations along the Cape coast, Hertling, as a part of her study, included a study of marram on the German Frisian Island of Sylt [1]. The conclusions of our studies are that the marram grass introduced to the Cape was uniform genetically, had the mycorrhizal fungi as mutualistically beneficial partners as a defence against nematode pathogens as in Europe [10,11], but did not set an abundance of seed. Moreover, it appeared that marram grass might only have been introduced once from a single locality in southern Europe. Other pioneer stabilising species indigenous to our region were also studied [12,13] but it was concluded that marram grass was the best dune stabiliser. We also concluded that there was no chance of it becoming invasive in this country as it has in other countries such as the USA [14] Australia and New Zealand [15], as long as new material was not introduced into South Africa from abroad. New introductions should not be made as the grass may set seed and then become invasive.

### 1.2. South African Legislation

The Conservation of Agricultural Resources Act (CARA) (Act 43 of 1983) regulations came into effect in 1983, listing 54 species requiring management strategies to be implemented by the landowner, with the level of management intervention depending on the category assigned to the species. When the Act was amended in 2001, the number of alien species listed had increased to 198 species. The guidelines for listing alien species have been further clarified according to the IUCN classification of alien taxa [20].

Under the Act, invasive alien species were regulated through regulation 15, where invasive alien species are classified into three categories. Category 1 species are weeds that are of no value, Category 2 are recognised weeds which have commercial value and Category 3 are those which are ornamental but provide no commercial value [21]. The 2001 list did not list marram grass as a weed/invasive alien species but as a Category 3 species. Bromilow [22], an authority on South African problem plants and weeds, states that proposals were made to list marram grass as a Category 2 species in the revised CARA that may be grown under permitted conditions in demarcated areas. 

Because of this weed listing of marram grass, a permit is required to use the species in the Western Cape to stabilise dunes in problem areas. It should be noted that it does not apply generally to South Africa, as marram grass is a dune species confined mainly to the Western Cape. Unfortunately, as researchers, we did not carry the findings over to advise or inform the authorities and practitioners on the safe use of the grass in South Africa. This may have resulted in the complete removal of marram grass from the list.

### 1.3. Dune Succession on the South-Eastern Coast of Africa

The vegetation of coastal dunes may be characterised by sequential stages of vegetation types or zones. The foredunes are often dominated by single plant species [23,24]. In the Eastern Cape, we found that there were often many pioneer species that will colonise and stabilise foredunes [25,26]. The result is hummock dunes or linear foredunes with different dominant species either of grass, herbaceous or succulent pioneer plants. In other cases, a combination of two or more pioneer species may be responsible for the stabilisation of sand and the build-up of foredunes [26]. The climax in the successional process is Coastal Scrub or Coastal Forest so there may be a number of pioneer pathways resulting in a single climax. In KwaZulu-Natal, the process was different in that the pioneers were restricted to one or two species, and the subsequent successional stages were more diverse, leading to more diverse types of Coastal Forests as the climax communities [25,27]. 

### 1.4. General Aim of this Study

For rapid stabilisation of dune systems, it is important to use the most efficient plant species for each situation. For this reason, rehabilitation or restoration practitioners favour the use of marram grass on Cape dunes, as it is the most useful plant to stabilise sands that are encroaching on development or endangering the erosion of built structures [8,26,28]. For this reason, there is still an interest in using marram grass in dune stabilisation programmes. 

In order to strengthen the case for marram as a useful stabilising species we therefore in this paper investigate:

(1) Does marram grass persist at all sites where it is planted without human intervention (i.e., has it become naturalised)?
(2) If marram grass spreads from sites where it has been planted to neighbouring areas, is this simply a case of natural dispersal or has it become actively invasive into other communities?
(3) Does marram grass in South Africa differ in its ecology from marram grass that is invasive elsewhere in the world?
(4) Does marram grass need to be listed under South Africa’s regulations on biological invasion?
(5) What is the environmental impact of marram on coastal environments?

In 2016, we were requested by the Municipality of Cape Town to carry out a Risk Assessment to identify potential risks in planting marram grass on dunes and beaches within the City of Cape Town [29]. The details of the Risk Assessment are given in that report, and it is not applicable to be elaborated upon in this paper.

To add weight to our Risk Assessment it was decided to investigate the current state of marram grass in the Eastern Cape. This area could be surveyed rapidly, and a large amount of data is already available for the coastal dunes as collected in the 1980s. This general aim was to sample relevés in a number of accessible sites in the Eastern Cape in order to measure the abundance of the marram grass and other species. Thus, within these sites we would be able to show how marram is distributed and how it interacts with other species in the dune system. This would give an indication of the natural position that marram grass has assumed in the dynamics and succession in the dune environment. Thus, marram grass may in the future be accepted as a non-invasive alien plant species.

## 2. Method

### 2.1. Coastal Sampling in the 1980s

From 1981 to 1984, Lubke and Avis [25] undertook extensive studies of the coastal environment of the Eastern Cape Coast, in terms of the ecology and the floristic composition and plant species abundance. Samples were collected at different sites whenever the coast was visited and numbered chronologically, but the relevés were later ordered and renumbered from Kei Mouth to Cape St Francis [25,30]. This was the full range of marram grass, which is not found farther north on the coast, probably due to its adaptation to more temperate conditions, usually with ample winter rainfall. Sites were not selected randomly but all sites that were visited on the coast were initially searched for marram grass, and all were sampled. Thus, there was no bias in the sampling as all marram sites were sampled. These became the reference sites for the 2017 sampling as outlined below.

At each site visited, the vegetation was sampled in relevés, and plant species were scored according to the Braun Blanquet approach (BB) of cover abundance [31,32,33,34]. These values were later converted to a numerical scale of 0 to 9. The plots were 10 × 10 m in dune or dune slack sites and rectangular plots of 5 × 20 m, where the sample was along a linear margin of dune scrub or thicket. All species were recorded, but little other data were recorded for each site. In all, 152 relevés were sampled along these sites on the dunes and 191 species were recorded for the study.

**Figure 1:**
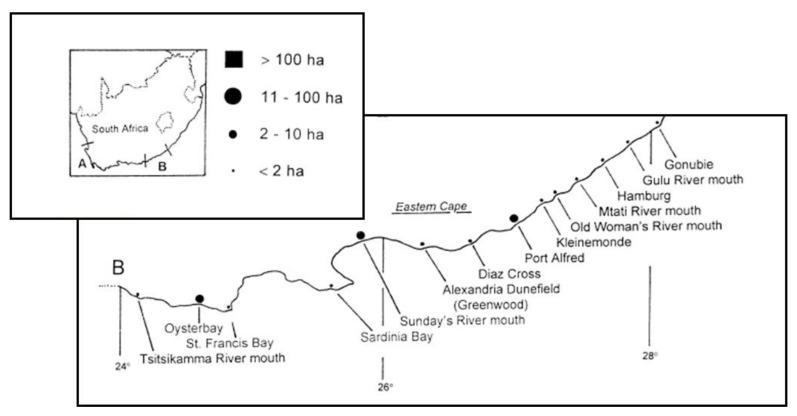
Distribution of *Ammophila arenaria* (marram grass) in the Eastern Cape, area B. Size of the dots shown in the map below indicates the area planted in hectares, see scale above. There was no area of >100 ha in this region (modified from Hertling [1]).

These data were used to extract information about where marram grass was recorded. In addition, the sites of historic information on the plantings of marram were noted from studies by Hertling and Lubke [1,8,16], (Figure 1 and Table 1).

### 2.2. Dune System Sampling in 2017

Within the distribution range of marram grass in the Eastern Cape from Tsitsikamma to Gonubie, 0.21 possible sites for sampling in 2017 were selected (Table 3). On 9 November 2017 and 12 November 2017, a number of these sites were visited to assess the presence and the distribution of the marram grass in these areas. 10 × 10 m quadrats were sampled for the cover-abundance of all species present in the pioneer zone, in sites where secondary colonisers were present and finally in areas where the coastal scrub species were present. In this way, it was envisioned that the change in abundance of the pioneers could be rapidly recorded from the pioneer zone through to the scrub zone. At each of the sites sampled in November a range between two and six relevés were recorded where marram grass was present at that site. If marram was no longer present in any of the former sites, they were not re-sampled. Twenty-five relevés were sampled in nine of the sites stretching from Hamburg to Oyster Bay (Table 3). Where marram was found to be present, we looked in adjacent sites up to 1km away for other sites where marram could occur in order to record these sites as well.

The presence and abundance of marram grass provide information on whether the grass has persisted in the area and is spreading or at least holding its own in the dune environment. The relevé cover-abundance data provides additional information as to how the species is functioning in the dune ecosystems. It would be expected that an invasive species would persist in the region where it was planted and if it is competitive and liable to oust indigenous species it would show an increase in the successive stages in the dune succession, even persisting into the climax vegetation as it does in the forest on the US west coast ([6]. On the Braun–Blanquet cover–abundance scale the highest value is 5 for 75 to 100% cover and this is regarded as dominant which is referred to in the results below.

Within the range of occurrence of marram grass in the Eastern Cape 33 of the original relevés sampled in the 1980s were chosen according to whether they were:

- Dune pioneer communities of hummock or foredunes (20 relevés)
- Coastal Scrub communities (12 relevés) and
- Dune Slack Community. (One relevé).

Of the currently 2017 sampled sites, the 36 relevés sampled on the young dunes could be divided into two communities:

- Early Dune pioneer communities (Dunes 2017) (25 relevés) and
- Early Coastal Scrub communities (11 relevés).

### 2.3. Data Analysis

When combined there were 69 relevés and 64 species which occurred in two or more of the relevés. The BB data were transformed into numerical values where absent = 0, r = 1; + = 2, 1 = 3; 2 m = 4; 2 a = 5; 2 b = 6, 3 = 7; 4 = 8; 5 = 9. In this way, a 69 × 64 numerical matrix was obtained for quantitative analysis. 

These data were then analysed with Detrended Correspondence Analysis (DECORANA) to simplify the data and show the relationships of the samples (relevés) and species in a 2-dimensional scatter diagram [35]. This technique is particularly useful to show the continuous change between communities in a dynamic system such as mobile dune systems.

## 3. Results 

### 3.1. The Disappearance of Marram Grass

Our first major observation was that marram grass was never found in areas where it had never been planted. A thorough examination of sites all along the coast verified that marram had not moved into new dune sites. The grass would have to seed prolifically to invade new sites and there was little evidence of seed production. Thus, our initial observation was that marram grass grows only in sites where tuffs of the grass have been planted. It can proliferate in these sites under favourable conditions, i.e., a steady supply of sand.

The sites that were sampled and the pioneer species recorded are listed in Table 3. Furthermore, of significance in the investigation of the potential of marram grass to invade is that there are four sites where marram grass is no longer present. In these areas, marram had been planted by hand as tufts often over many hectares of mobile dunes. If we examine the demise of marram grass in these four sites, we can note the following:

- Site 1—Gonubie, East London: The site was an eroding rear dune planted in 1990s by the local Municipality. This site was not suitable as there was not a build-up of sand and the rainfall is limiting in the winter and rather sporadic. Thus, marram was out of its range at this site and had disappeared.
- Site 5—Old Woman’s River—was planted in 1980 by the Department Of Forestry. The area to the west of Old Woman’s River mouth is the coastal sweep of Waterloo Bay, which is bordered by a mobile dune field subject to periodic erosion and accretion. It is likely that the erosion of sand under high tide storm conditions or with high winds has resulted in the loss of the grass.
- Site 6. Fish River Mouth—was planted in 1973 by the Department Of Forestry as part of their plan to stabilise all the mobile dunes. The dunes at the mouth are eroded periodically and the grass could not survive under these conditions. Currently, the foredunes are stabilised by the indigenous pypgras (*Ehrharta villosa*).
- Site 20—St Francis Bay, Main Beach. The mobile headland bypass dunefield was initially planted in 1917–1924 by Department Of Forestry. Developers also stabilised the area in the 1970s. The marram grass has completely disappeared as the beach and dunes have eroded due to the cut-off of sand supply.

**Table 3:** Sample sites of marram grass in the Eastern Cape and some results recorded in 2017.

No.	Site	Number of Rel. Sampled	Date	GPS Coordinates	Pioneer Species Recorded (a Blank Cell Indicates a Site Not Resampled in 2017)	Habitat Type	Remarks
1	Gonubie, East London	0		32°56′12.69″ S; 28°1′53.90″ E	No *Ammophila arenaria* present	Eroding rear dune	Planted in 1990s by Municipality
2	Gulu River Mouth	0		33°7′10.34″ S; 27°43′45.22″ E		Foredunes and Dune Field	Planted in 1978–1982 by Department Of Forestry
3	Hamburg	6	12/11/2017	33°16′56.33″ S; 27°29′13.40″ E	*Ammophila arenaria* *Ipomoea pescaprae* *Sporobolus virginicus* *Scaevola plumieri*	Foredunes and dune field	
4	Mtati River Mouth	3	12/11/2017	33°25′17.32″ S; 27°15′22.62″ E	*Ammophila arenaria* *Ipomoea pescaprae* *Ehrharta villosa*	Rear dunes above dune slack	Planted in 1978–1982 by Department Of Forestry
5	Old Woman’s River	0	5/11/2017	33°28′59.34″ S; 27°8′51.52″ E	No *Ammophila arenaria* present	Dune field	Planted in 1980 by Department Of Forestry
6	Fish River Mouth	0	November 2017	33°29′54.47″ S; 27°7′59.21″ E	No *Ammophila arenaria* present	Dune field	Planted in 1973 by Department Of Forestry
7	Kleinemonde	8	12/11/2017	33°32′32.26″ S; 27°2′43.13″ E	*Agropyron distichum* *Ammophila arenaria* *Ipomoea pescaprae* *Sporobolus virginicus* *Scaevola plumieri*	Dune blow-out	Planted in 1970s and 1980s by local farmer
8	Port Alfred	5	12/11/2017	33°36′13.65″ S; 26°53′58.03″ E	*Ammophila arenaria* *Ipomoea pescaprae* *Agropyron distichum* *Ehrharta villosa* *Arctotheca populifolia*	Dune field	Planted in 1883 by Harbour Authorities
9	Kenton-on-Sea	3	12/11/2017	33°41′28.73″ S; 26°39′59.42″ E	*Ipomoea pescaprae* *Ehrharta villosa* *Arctotheca populifolia*	Linear dunes	
10	Diaz Cross, Kwaaihoek	0		33°43′3.94″ S; 26°37′24.57″ E		Dune field and migrating dunes	
11	Alexandria Dunefield	0		33°43′12.63″ S; 26°13′59.92″ E		Dune field and migrating dunes	Planted in 1981 to 1991 by Department of Forestry
12	Sundays River Mouth	0		33°43′4.96″ S; 25°51′49.34″ E		Dunes at River mouth	Planted in 1960s–1977 by Department Of Forestry
13	Port Elizabeth Harbour	0	2012	33°57′54.31″ S; 25°38′37.45″ E		Migrating dunes	Planted in 1890s–1990 by Harbour Authorities
14	Port Elizabeth Summerstrand Beach	3	9/11/2017	33°59′21.39″ S; 25°40′28.36″ E	*Sporobolus virginicus* *Agropyron distichum* *Ehrharta villosa* *Arctotheca populifolia* *Gazania rigens*	Foredunes and dunes	Planted in 1890s to 1990 by Department of Forestry
15	Cape Receife	0	9/11/2017	34°1′38.99″ S; 25°41′1.49″ E		Hummock dunes	Planted in 1976–1977 by PE Municipality
16	Sardinia Bay	3	9/11/2017	34°2′3.10″ S; 25°29′45.49″ E	*Agropyron distichum* *Ammophila arenaria* *Ehrharta villosa*	Steep dunes	
17	Jeffreys Bay: Paradise Beach West	3	9/11/2017	34°6′45.40″ S; 24°52′46.54″ E	*Ammophila arenaria* *Agropyron distichum* *Ehrharta villosa* *Arctotheca populifolia*	Foredunes	
18	Kromme River Mouth: East Bank	0		34°8′27.03″ S; 24°50′32.45″ E		Dunes	
20	St Francis Bay- Main Beach	0	9/11/2017	34°9′55.98″ S; 24°49′54.07″ E	No *Ammophila arenaria* present	Foredunes	
21	Oyster Bay	2	9/11/2017	34°10′22.96″ S; 24°39′18.67″ E	*Ammophila arenaria* *Agropyron distichum* *Ehrharta villosa*	Foredunes	Planted in 1917–1924 by Department Of Forestry
**Total No. of Relevés**		**36**	**Total No. of Pioneer spp.**		***8***		
**Dune Pioneer Relevés**		**11**	**Mean No. of Pioneer spp.** **Per site**		***2.1***		
Early Coastal Scrub Relevés		25	Number of sites (regions) where marram has disappeared		4		

### 3.2. The Presence and Abundance of Marram Grass in the Sites Sampled

In no cases where marram grass had been planted did we find the grass in nearby adjacent sites. In other words, marram may persist at some sites but there is no evidence of it having moved from these sites to adjacent favourable sites.

**Figure 2:**
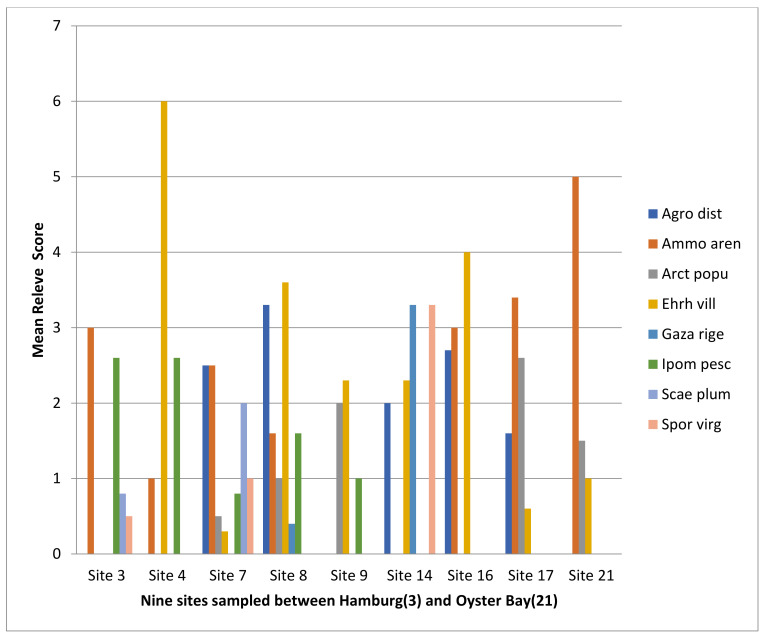
Abundance of *Ammophila arenaria* (marram grass) and seven other pioneer dune species sampled in 9 sites along the Eastern Cape coast. (Site 3 = Hamburg; 4 = Mtati Mouth, 7 = Kleinemonde; 8 = Port Alfred; 9 = Kenton; 14 = Port Elizabeth, Summerstrand; 16 = Sardinia Bay; 17 = Paradise Beach; 21 = Oyster Bay. For full names of species, see Table 3 above and in the text.).

Three to five dune pioneer species were recorded at each site, as shown in Table 3 and Figure 2, which show the mean abundance of the species as recorded in the 10 × 10 m relevés at each of the nine sites that were sampled. The eight pioneer species recorded in the sites sampled are:

*Agropyron distichum* (Thunb.) Beauv.—sea wheat

*Ammophila arenaria* (L.) Link.—marram grass

*Arctotheca populifolia* (Berg.) T. Norl.—sea pumpkin

*Ehrharta villosa* Schult. F. var. *maxima* Stapf.—pypgras

*Gazania rigens* (L.) Gaertn. var. *uniflora* (L.f.) Roessler.—dune daisy

*Ipomoea pes-caprae* (L.) R.Br. var. *brasiliensis* (L.) van Ooststr.—dune morning glory

*Scaevola plumieri* (L.) Vahl. –seeplakkies

*Sporobolus virginicus* (L.) Kunth.—brakgras.

The mean number of pioneers in the sites sampled was two (Table 3) and the highest number of pioneer species was found in site 7 (Kleinemonde) with seven species, only *Gazania rigens* being absent at that site. The number of pioneer species in the relevés ranged from one to five and there was a range of abundance in the relevés (Figure 2). There were also a few secondary species in some of the relevés. 

It will be noted from Figure 2 that marram grass does not dominate the dune environment although in Oyster Bay (21), Paradise Beach (17) and Hamburg (3) dunes it is the most abundant dune pioneer. An indigenous grass pioneer species *Ehrharta villosa* (pypgras; is dominant in Mtati (4), Port Alfred (8), Kenton (9) and Sardinia Bay (16) dunefields.

### 3.3. The Role of Marram Grass in the Normal Dune Successional Process

**Figure 3:**
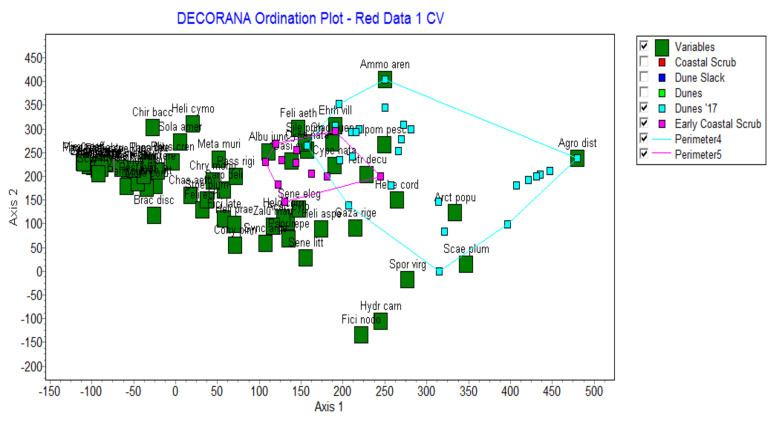
Scatter plot of all the 64 species of the coastal dunes in the Eastern Cape sites on the first two axes produced by Detrended Correspondence Analysis. See Table 3 for species names listed in full.

The results above show that marram grass is present but never dominates the pioneer dune communities. An analysis of the quantitative data shows that when a scatter diagram of the 64 species is produced on the first two axes of Detrended Correspondence Analysis, the pioneer species are widely dispersed, mainly on Axis 2 (Figure 3). In contrast, the shrub species characterising the coastal scrub are tightly clustered to the left of the figure. The perimeter of the relevés of the dune pioneer communities and the early coastal scrub communities are also shown in this figure to relate the species to the communities. 

**Figure 4:**
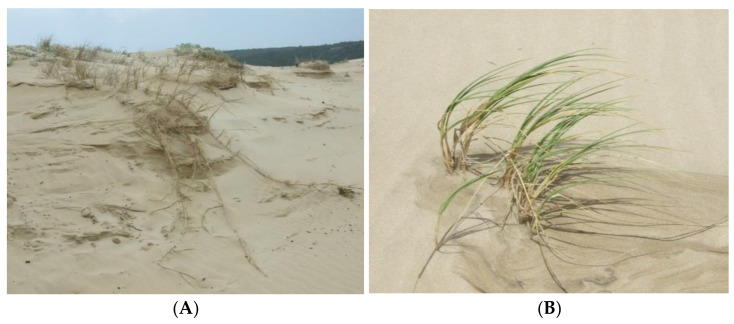
(**A**) Clumps of *Agropyron distichum* (sea wheat) on a wind eroded dune at Kenton-on-Sea. Note the exposed elongated rhizomes. (**B**) A young sea wheat plant in the wet sand.

The pioneer species that is usually closest to the shore is *Agropyron distichum* (sea wheat). It produces seedlings in the damp sand where the seeds germinate, and it also is dispersed vegetatively by elongated rhizomes (Figure 4). Note that in Figure 3 *Agropyron distichum* is the species on the far right of the scatter diagram, sites that are closest to the high tide mark. It is not possible to read the names of all species on the figure but the important pioneer species (Table 3) stand out from the mass of other species on the left of the diagram.

**Figure 5:**
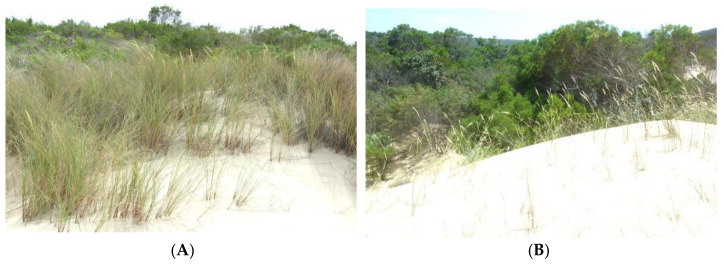
(**A**) *Ammophila arenaria* (marram grass) at Oyster Bay being replaced by shrubs with coastal scrub in the distance. (**B**) *Ehrharta villosa* (pypgras) at Hamburg on the rear dunes, where it is replaced by shrubs such as *Passerina rigida* (dune string).

At the top of the scatter diagram is marram grass, and the pioneer species on the bottom figure are three other pioneer species (*Arctotheca populifolia*—sea pumpkin; *Scaevola plumieri*—seeplakkies; and *Sporobolus virginicus*—brakgras). Thus, from these different pioneer communities there will be an invasion of secondary coloniser species into the pioneer zones as the sand is stabilised ultimately all being replaced by the climax Coastal scrub species (Figure 5).

**Figure 6:**
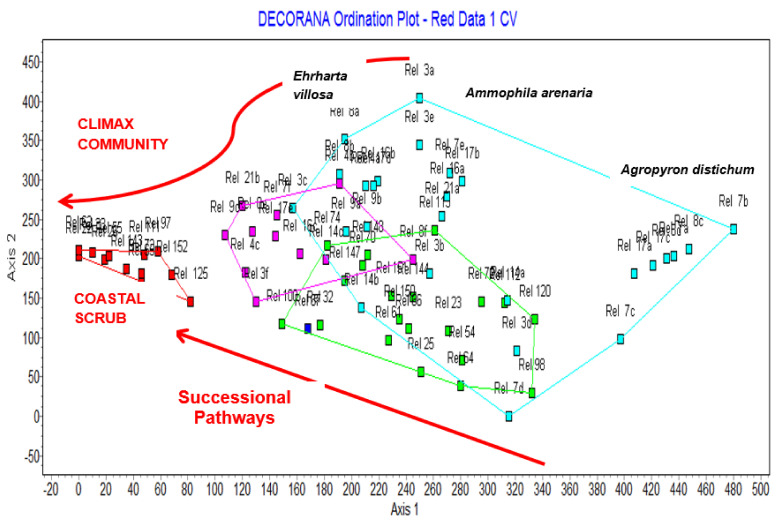
Scatter plot of all the 69 relevés of the coastal dunes in the Eastern Cape sites on the first two axes produced by Detrended Correspondence Analysis. The four communities are shown, and their perimeters designated. The arrows show that the successional pathways and some dominant pioneer species are positioned where they are most abundant. The three pioneer communities are dominated by the three species indicated: early coastal scrub—*Ehrharta villosa* (pink); dune pioneer communities of hummock or foredunes—*Ammophila arenaria* (green); and early dune pioneer communities—*Agropyron distichum* (blue). The communities are shown roughly as closest and farthest distance from the shoreline from right to left.

Thus, it would appear that the pioneer dune communities all behave similarly in the dune successional series. The pioneer species colonise the bare dune sands, stabilise them and secondary species then migrate to other sites in the modified environment. The secondary species are more suited to the stabilised sand and the shady conditions. In the case of marram, the grass requires actively mobile sands to thrive. At a later stage, there are shrubs species colonising the area and final some trees of the climax Coastal Scrub. This is shown in the scatter diagram of the 69 relevés produced on the first two axes of Detrended Correspondence Analysis (Figure 6). 

This figure can be compared with Figure 3 to see the positions of relevés that are dominated by different pioneer species. The species dominant in the different relevés have been indicated in the diagram; for example, on the extreme left of the figure, relevé 7b, which is closest to the shoreline, has a cover of about 5% of only *Agropyron distichum* and no other species are present in the relevé. Other relevés in the region (on the right of Figure 6) also have a high cover value for this species. The successional pathways are shown in the figure as the pioneer communities move towards the climax community of coastal scrub (on the left of Figure 6), which will ultimately become a dense thicket.

## 4. Discussion

### 4.1. The Extent of Marram Grass in the Sites Sampled

Avis [4,25] carried out extensive studies on the stabilisation of the dune systems by the Department of Forestry who had a policy at that time of stabilising all mobile dunes systems. After this extensive dune planting, hundreds of hectares had been stabilised and it was common to record areas of 10ha or more that were being stabilised with marram grass. Avis [25]) records sites in the Alexandria dune fields, for example, that were being planted with fields of marram grass, as illustrated in the pamphlet of Lubke and Avis [28]. In this pamphlet, we suggested the best ways that mobile sands could be stabilised. Thus, compared with the extent of the stabilised areas using marram grass in the 1970s and 1980s, we expected some of the vast sites still to be present in our 2017 survey. However, this was not the case and there was never a dune system in the later survey more than 1ha in extent of marram grass. Mostly, it took careful surveillance of the dunes to find the marram grass when present, as it often formed an association of pioneer dune species. Hertling [1] in her sampling in the few years prior to this date (1995–1997), also recorded the extent of the marram at the various sites, which had diminished from when Avis [4] had sampled them in 1985 to 1989. Thus, one can conclude that the grass has become reduced in the area of the mobile dune systems where it was planted.

### 4.2. Is Marram Grass Invasive in the Coastal Environment in the Cape?

We have established that marram does not occur except where it was planted, and at many sites marram simply disappears (Table 3). Where it is still present at those sites it is never dominant but forms a natural part of the dune system. Moreover, there is no evidence of the spread of the grass from these sites to adjacent areas. Where the marram grass does still occur, it is at most a codominant with other pioneer species (Table 3 and Figure 3). The marram grass has an ecological impact that is favourable in the successional process, as shown in the quantitative analysis (Figure 4, Figure 5 and Figure 6). Thus, the socio-economic impact of marram grass is that it is an advantage in dune stabilisation on the Cape coasts of South Africa. The grass behaves differently in South Africa compared to other parts of the world as explained in the requirement for the growth and dispersal of the grass below. Because of these findings, there is no evidence for marram grass to be listed as an invasive alien species in South Africa. 

### 4.3. The Requirements for the Favourable Growth and Dispersal of Marram Grass 

In order to be a successful pioneer in the dune environment, the plant must be able to get to the bare uncolonised sands either through seeds or vegetative propagules. The seeds need to be able to germinate under these conditions and become established [26,27,36]. With dune pioneers such as the indigenous pioneers *Scaevola plumieri*, *Arctotheca populifolia* (dicots) and *Agropyron distichum* (grass) (Figure 5B), we have recorded young seedlings in the wet sand after heavy [26]. Other species such as *Ehrharta villosa* are more likely to colonise sites using their extensive horizontal rhizomes (Figure 4). This species has stabilised the east bank of the Bushmans River near the mouth, which has been the subject of an intensive study of the interaction of the dune and the estuary system by. Lubke and Webb [37], who found no evidence of marram in this new dune system, which has all the favourable habitat features for the growth of the grass [38]. It has never been planted at this site. In the Alexandria dune field to the west, many hundreds of hectares were planted. None of those marram plants have been washed away by high tides nor been carried east with the long shore drift to colonise this area, as would be expected if rhizomes were able to withstand exposure to sea water.

Marram grass does not set seed in the Cape and does not have extensive rhizome systems so it is holding on in areas where it has been planted as tufts [28]. In local areas, it may show a limited dispersal, by drifting of rhizome fragments in the wind, and is still found as a successful pioneer in several sites in the Eastern Cape as has been recorded above. Knevel [12] showed that rhizomes were unable to grow after prolonged submergence in seawater, so it is unlikely to be dispersed by wave action and tides except over short distances. 

Recent studies in New Zealand by Hilton [15] have found that there, marram grass can grow following immersion in seawater, but in this case, large rhizome pieces were used [39]. The results from the study showed that only a single fragment is required to establish a new colony of marram grass, but an invasion from a single dispersal event is highly unlikely. However, factors such as foredune scarping and rivermouth migration could result in a high supply of rhizome fragments [39]. We have not attempted to replicate these experiments with our ‘Cape’ marram grass form.

Marram also requires a cool temperate climate and winter rainfall, so its distribution is limited to the Cape areas, as shown by Ulla Hertling in her extensive survey of the Cape dunes [8,16].and also in the native European situation in German dunes [1].

**Figure 7:**
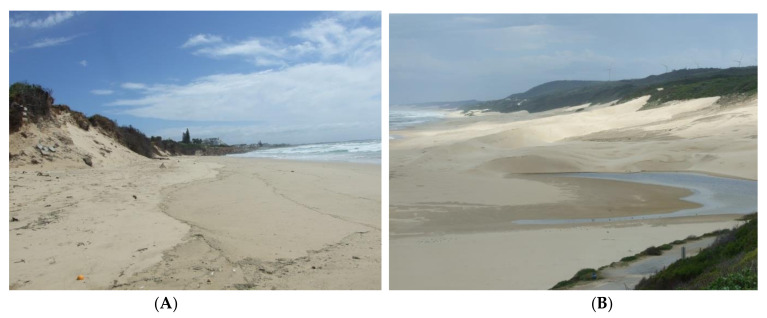
(**A**) St Francis Bay Main Beach that has completely eroded due to inappropriate stabilisation of the dune field that provided the sand supply. Holiday homes are in danger of collapse, as they are positioned just above the eroding dunes shown here (see Lubke 1988). (**B**) Maitland River Mouth where marram grass has never been introduced but any dune pioneers would have little chance of becoming established due to the exposed nature of the undisturbed dunes and the high wind velocities achieved in this area.

Thus, marram grass has disappeared from sites in Gonubie where it was planted in the extreme east of its Cape range. Extreme climates of high winds and dry periods have also limited its spread in other areas. It has all but disappeared from the Alexandria dunefield where hundreds of hectares were planted in the 1970s and 1980s. Lubke [40] sampled the eroding beach at St Francis Bay in the 19870s and 1980s in a study that showed the progressive erosion of the dunes over a 10-year period. He also forecast the complete loss of the foredunes in time. The marram grass has completely disappeared as the beach and dunes have eroded (Figure 7A.) due to the cut-off of sand supply by the stabilisation of the Headland Bypass Dune Field that supplied sand to the beaches on the eastern side of the headland (peninsular) [40]. Marram grass was never planted at Maitland River Mouth (Figure 7B), but pioneers are unlike to survive on these wind-swept dunes. None of the indigenous pioneers have been successful on these undisturbed natural dune fields.

**Figure 8:**
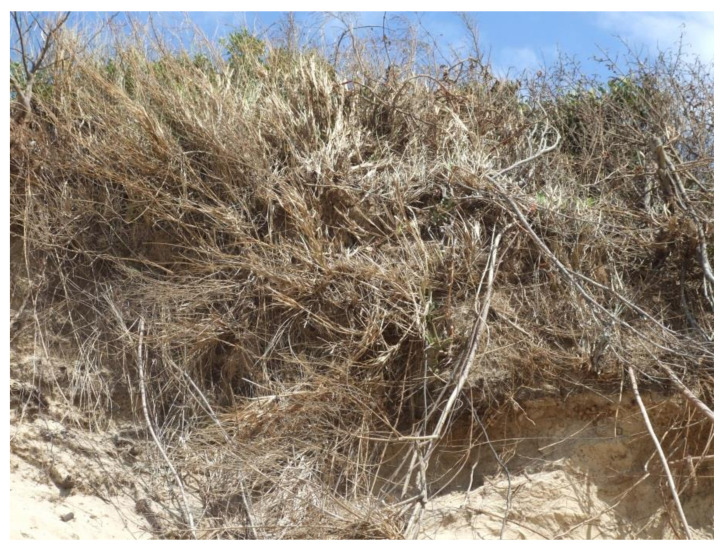
Moribund *Ammophila arenaria* with its erect rhizomes that grow up as the drift sands are deposited.

Marram grass also has a limited life in situations where sand does not continue to accumulate. The rhizomes grow vertically, and it is thus able to withstand burial as observed by Maun [24]. The nature of the rhizomes and their growth form in the mature state is shown in Figure 8.

### 4.4. The Coastal Dune Environment in the Eastern Cape

Lubke and Avis [26] described the coastal dune system and the succession of plants from pioneers through to the climax dune thicket. This Clementsian-type model that we proposed holds for much of the Eastern Cape, and the results we have achieved in this study as shown diagrammatically (Figure 2, Figure 3 and Figure 6) also conform with this type of succession. The feature of the successional process is that many pioneer species, herbs, shrubby succulents, and grasses may become established in the mobile bare sands individually or combined with several other pioneers. Once stabilised, the hummock or linear dunes are colonised by a few secondary colonisers and some of the well-adapted shrubs such as *Passerina rigida* (Figure 5) and *Stoebe plumosa* also colonise the area [25,26]. By recording data for almost 20 years at a single site near Kleinemonde, we found that more climax species (trees and shrubs) become established over time, so that Coastal Thicket is the climax vegetation [36].

What is important to this study is the place of marram grass in the dune succession and its interaction with the indigenous species. As we have noted (Figure 6) and from previous studies on dune succession [7], there are multi-directional pathways and marram grass fits naturally into this system. It now behaves identically to. the indigenous species as a dune pioneer. The mobile dunes in the De Mond Nature Reserve were planted by the Department of Forestry to stabilise the sands at the river mouth and prevent the closure of the mouth. Some 30 years later we found Dune Fynbos had now replaced the mobile dunes and marram grass was only present in the more recently planted areas near the river mouth [19].

### 4.5. Consequences of This Study in the Use of Marram Grass in Dune Stabilisation Projects

**Figure 9:**
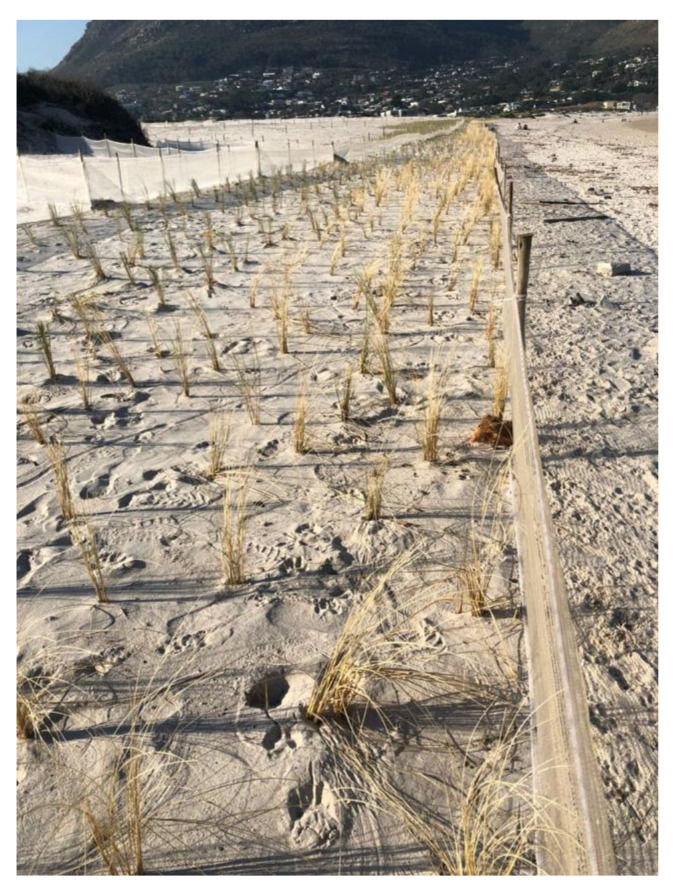
Marram grass planted at Hout Bay in 2017, near Cape Town to prevent sand from blowing onto the car park at the harbour (photo: Ted Avis). This project won the South African Landscapers Institute’s (SALI 2020) top award for excellence in landscaping, out of more than 100 projects submitted.

As a result of this study and the Risk Assessment produced by CES, the City of Cape Town was given permission to plant marram grass as a dune stabiliser in situations where dune sands were problems in the built environment [29]. Tufts of marram grass were recently recorded as have been planted at Hout Bay. Ted Avis provided a photograph of this study in 2017 (Figure 9). The success of this project indicates the advantage of using marram grass to stabilise the dune systems, when necessary, along the eastern, southern and western Cape coasts.

## 5. Conclusions

Marram grass in the Cape dune systems does not persist in the climax vegetation. It occurs in some cases along with other pioneer species, but in many cases, it just disappears, as the stabilised sand is unfavourable for the grass to persist in the stable dune system. Thus, we can conclude that marram grass is a non-invasive species that can be successfully used in dune stabilisation should an assessment of the situation indicate that stabilisation of the dune system is required. Moreover, in the dynamics of dune succession, marram grass behaves identically to the indigenous species as a dune pioneer.

## Figures and Tables

**Table 1:** Planting of marram grass (*Ammophila arenaria*) in the Eastern Cape. (See Figure 1 for location of the sites) (Information from Hertling [1], where known).

Site	Total Area Planted	Time of Planting	Source of *A. arenaria* (*Where Known*)	Plantings Carried Out By
Oyster Bay/ St. Francis Bay	358 ha	1917–1924	–	Department Of Forestry
Oyster Bay/ St. Francis Bay	–	since 1964	–	Department of Forestry
Sardinia Bay	–	–	–	–
Port Elizabeth	–	1890s–1990	Western Cape	Harbour Board, Divisional Council, Department of Forestry
Port Elizabeth. Cape Recife	–	1976–1977	own material	Port Elizabeth Municipality
Sunday’s River mouth	4 ha	1960s–1977	–	–
Alexandria Dunefield— Several areas	ca. 500 ha	1981–1991		Department of Forestry
Dias Cross, Kwaaihoek	–	–	–	–
Port Alfred	Sowing experiment	1883		Port Alfred Harbour Authorities
Kleinemonde	–	–	–	–
Fish River Mouth—Fish Point	0.8 ha	1973		
Old Woman’s River Mouth		1980		Department of Forestry
Mtati River Mouth		1978–1982		Department of Forestry
Hamburg	–	–	–	–
Gulu River Mouth	–	1978–1982		Department of Forestry
Gonubie, East London	–	1990s	Alexandria, De Hoop	Gonubie Municipality

**Table 2:** List of publications on *Ammophila arenaria* (marram grass) and related dune pioneers investigated as part of the Invasive Grass species study (INVASS).

Author(s) and Date	Title of Publication	Source
Hertling, U.M., 1997 [1]	An autecological study of *Ammophila arenaria* and alternative indigenous species used in dune stabilisation on the Cape coast.	Unpublished Ph.D., Rhodes University, Grahamstown.
Hertling, U.M., Lubke, R.A., 1999 [16]	Indigenous and *Ammophila arenaria*-dominated dune vegetation on the South African Cape coast.	*Applied Vegetation Science*, 2: 157–168.
Hertling, U.M. and Lubke, R.A. 2000 [17]	Assessing the potential for biological invasion –the case of *Ammophila arenaria* in South Africa.	*South African Journal of Science*, 96: 520–527.
Knevel, I.C. 2001 [12]	The life history of selected coastal foredune species of South Africa.	Unpublished Ph.D., Rhodes University, Grahamstown.
Knevel, I.C., Venema, H.G. and Lubke, R.A. 2002 [13]	The search for indigenous dune stabilizers: Germination requirements of selected South African species.	*Journal of Coastal Conservation*, 8: 169–178.
Knevel, I.C; and Lubke, R.A. 2004 [18]	Reproductive phenology of Scaevola plumieri; a key coloniser of the coastal foredunes of South Africa.	*Plant Ecology*, 175: 137–145.
Knevel, I.C., Lans T, Menting F.B.J, Hertling U.M. and Van der Putten W.H. 2004 [10]	Release from native root herbivores and biotic resistance by soil pathogens in a new habitat both affect the alien *Ammophila arenaria* in South Africa.	*Oecologia* 141: 502–510.
Knevel, I.C., R.A. Lubke and Van der Putten, W.H. 2005 [11]	Release from native root herbivores and biotic resistance by soil pathogens in a new habitat both affect the alien *Ammophila arenaria* in South Africa	In: Herrier J.-L., Mees, J., Salman, A., Seys, J., Van Nieuwenhuyse, H. and Dobbelaere I. (Eds). *Proceedings ‘Dunes and Estuaries 2005′—International Conference on Nature Restoration Practices in European Coastal Habitats*, Koksijde, Belgium. pp. 179–189.
Lubke, R.A. and Hertling, U.M. 1999. [8]	Use of *Ammophila arenaria* for dune stabilisation in South Africa and its current distribution—Perceptions and problems.	*Environmental Management*, 24: 467–482.
Lubke, D.B. and Hertling, U.M. 2001 [19]	The role of European marram grass in dune stabilisation and succession near Cape Agulhas, South Africa.	*Journal of Coastal Conservation*, 7: 171–182.
